# Synergistic effects of Cyp51 isozyme-specific azole antifungal agents on fungi with multiple *cyp51* isozyme genes

**DOI:** 10.1128/aac.00598-25

**Published:** 2025-09-26

**Authors:** Masaki Ishii, Kazuki Ishikawa, Kazuhiro Mikami, Koji Ichinose, Atsushi Miyashita, Takashi Yaguchi, Tsuyoshi Yamada, Shinya Ohata

**Affiliations:** 1 Research Institute of Pharmaceutical Sciences, Faculty of Pharmacy, Musashino University 13214, Nishitokyo, Japan; 2 Faculty of Pharmaceutical Sciences, Teikyo Heisei University 370435 , Tokyo, Japan; 3 Graduate School of Medical Care and Technology, Teikyo University 70652, Hachioji, Japan; 4 Teikyo University Institute of Medical Mycology (TIMM), Hachioji, Japan; 5 Medical Mycology Research Center, Chiba University 118076, Chiba, Japan; 6 Asia International Institute of Infectious Disease Control, Teikyo University 13094, Tokyo, Japan; University Children's Hospital Münster, Münster, Germany

**Keywords:** dermatophyte, *Trichophyton rubrum*, Cyp51 isozymes, Cyp51A, azole synergism, azole antifungal, antifungal resistance, *Aspergillus*

## Abstract

Pathogenic fungi pose significant societal challenges due to limited therapeutic targets resulting from the eukaryotic nature of fungi. This limitation emphasizes the importance of enhancing susceptibility to inhibitors of Cyp51, a crucial enzyme in ergosterol biosynthesis targeted by azole antifungals. In Cyp51 isozyme deletion strains (Δ*cyp51A* and Δ*cyp51B*) of *Trichophyton rubrum*, the predominant dermatophyte species, we found that Cyp51B is essential for basal mycelial growth, while Cyp51A functions as an inducible isozyme associated with azole tolerance. Based on these differential functions, we hypothesized that each isozyme would show distinct susceptibility to azole antifungals. Our study demonstrated that most azoles exhibited increased antifungal activity against Δ*cyp51A*, while select agents demonstrated increased antifungal activity against Δ*cyp51B*. Remarkably, fluconazole, sulconazole, and imazalil exhibited relatively increased activity against Δ*cyp51A*, whereas prochloraz demonstrated increased activity against Δ*cyp51B*. Combining these isozyme-selective agents exerted synergistic effects against the wild-type strain and the parent *ku80*-knockout strain but not against individual Cyp51 knockout mutants. Our data revealed that the two Cyp51 isozymes can be selectively inhibited by different azole antifungals, resulting in a synergistic effect when combined. This synergistic effect was also observed on another fungal species, *Aspergillus welwitschiae*, which also has two Cyp51 isozymes. These data demonstrate that combining azole antifungals with different Cyp51 isozyme selectivities exerts synergistic effects against fungi possessing multiple Cyp51 isozymes. These findings advance antifungal therapeutic strategies by demonstrating that the combination of antifungals with different Cyp51 isozyme selectivities offers a promising approach for treating fungal infections, opening new avenues for isozyme-specific drug development.

## INTRODUCTION

Fungal infections represent a significant global health challenge, causing 3.752 million annual deaths (1). Among nonlethal fungal infections, dermatophytosis affects >10% of the world’s population and significantly influences patients’ quality of life through direct symptoms, such as itching and inflammation, and also potentially exacerbates conditions, such as asthma, and increases the risk of developing diabetic foot ulcers (2–4). The development of selective antifungal drugs is particularly challenging due to the similarity between fungal and mammalian cellular components (5–7), necessitating the discovery of both novel drug targets and approaches to existing molecular targets.

Combination therapy typically uses drugs targeting distinct molecules to exert additive or synergistic effects (8). Nevertheless, studies have demonstrated that combining drugs from the same class can also be effective. For instance, β-lactam antibiotic combinations have been reported to be effective both *in vitro* and in clinical settings (9–11) because they interact with multiple penicillin-binding proteins (PBPs) with varying affinities (12). When two β-lactams with different PBP preferences are combined, they can effectively inhibit multiple PBPs at lower concentrations. This principle suggests that pathogens with multiple isozymes of the same target enzyme might be susceptible to enhanced inhibition through combinations of drugs with distinct isozyme preferences. Although the synergistic effects of same-class combinations have been well investigated in antibacterial therapy (10, 11, 13), there is a lack of similar investigations with antifungal drugs.

Azole antifungals, the most widely used class of antifungal drugs, target the sterol 14α-demethylase enzyme Cyp51 in the ergosterol biosynthesis pathway (14). Although Cyp51 is encoded by a single essential gene (*erg11*) in *Candida albicans* and *Saccharomyces cerevisiae*, some filamentous fungi, including *Trichophyton* and *Aspergillus* species, possess multiple *cyp51* genes (14). For instance, *Trichophyton rubrum*, the most common causative agent of dermatophytosis, expresses both Cyp51A and Cyp51B (14). Nonetheless, the isozyme-specific activity profiles of existing azole antifungal agents remain largely unexplored. This critical knowledge gap hinders the development of more effective combination therapies.

We hypothesized that azole antifungals exhibit differential selectivities for Cyp51 isozymes, and that combinations of azoles with distinct isozyme preferences could improve sensitivities via synergistic effects. In this study, we generated *cyp51A* and *cyp51B* deletion strains in *T. rubrum* and demonstrated the isozyme selectivity of various azole antifungals. Our findings provide a foundation for developing novel combination therapies that exploit isozyme-specific vulnerabilities in pathogenic fungi.

## RESULTS

### Characterization of Cyp51 isozyme deletion strains in *T. rubrum*

**Fig 1:**
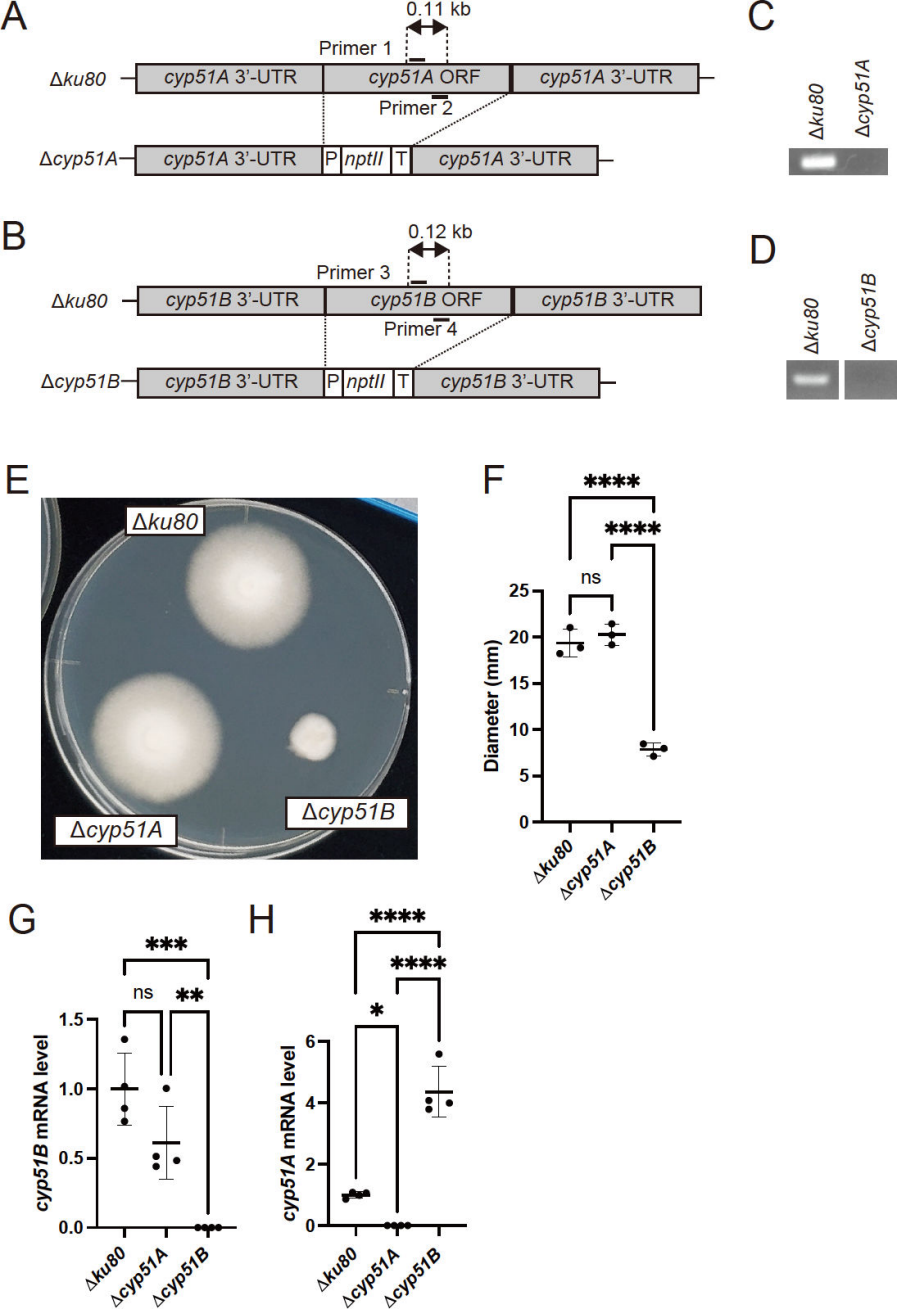
Generation and characterization of *cyp51A* and *cyp51B* deletion strains of *T. rubrum*. (**A**) Schematic of the *cyp51A* locus of WT and ∆*cyp51A* strains. (**B**) Schematic of the *cyp51B* locus of WT and ∆*cyp51B* strains. (**C**) PCR analysis of total DNA samples from the ∆*cyp51A* strain. The fragments were amplified using primer pairs (primers 1 and 2). Δ*ku80* was used as a control. (**D**) PCR analysis of the total DNA samples from the ∆*cyp51B* strain. The fragments were amplified using primer pairs (primers 3 and 4). Δ*ku80* was used as a control. (**E**) Mycelial growth of Δ*ku80*, ∆*cyp51A*, and ∆*cyp51B* on SDA at 28°C for 14 days. (**F**) Colony diameter of Δ*ku80*, ∆*cyp51A*, and ∆*cyp51B* strains on SDA at 28°C for 14 days. The bars represent the mean ± standard deviation (SD) of data obtained from three independent experiments (*n* = 3). ****P  <  0.0001. (**G and H**) Expression levels of *cyp51B* (**G**) and *cyp51A* (**H**) mRNA in Δ*ku80*, ∆*cyp51A*, and ∆*cyp51B* strains as determined by qRT-PCR. The fold change represents the gene expression level compared with that of Δ*ku80*. The bars represent the standard deviation of data obtained from three independent experiments (*n* = 4). *P  <  0.05; **P  <  0.01; ***P  <  0.001; ****P  <  0.0001.

We recently generated a strain deficient in *ku80*, which is involved in nonhomologous end joining, for efficient genetic recombination in *T. rubrum* (15). Based on that report, we generated deletion strains of individual Cyp51 isozymes (Cyp51A and Cyp51B) in the *T. rubrum* Δ*ku80* strain. The successful deletion of *cyp51A* and *cyp51B* was confirmed by polymerase chain reaction (PCR) using the primers depicted in Fig. 1A and B (Fig. 1A through D). The *cyp51A* deletion strain (Δ*cyp51A*) demonstrated growth comparable to that of the parent strain Δ*ku80*, consistent with our previous observations of mutants (15). In the earlier study, the insertion of a drug resistance gene into the 3′-UTR region of *cyp51A* resulted in reduced expression without exhibiting any visible growth defects in *T. rubrum* (15). Interestingly, the *cyp51B* deletion strain (Δ*cyp51B*) exhibited a significant mycelial growth defect (Fig. 1E and F), which was consistently observed in an independently generated deletion strain (see Fig. S1). This finding suggests that Cyp51B is essential for basal mycelial growth in *T. rubrum* under standard culture conditions.

Expression analysis revealed no compensatory upregulation of *cyp51B* mRNA when *cyp51A* was deleted, indicating that basal *cyp51B* mRNA expression is sufficient for normal growth in nutrient media (Fig. 1G). Nevertheless, *cyp51A* mRNA expression was upregulated in the *cyp51B* deletion strain (Fig. 1H). Moreover, exposure to azoles induced *cyp51A* mRNA expression but not *cyp51B* mRNA expression (15), suggesting that Cyp51A functions as an inducible isozyme that responds to insufficient ergosterol synthesis.

### Differential sensitivity of Cyp51 isozyme deletion mutants to azole antifungals

To gain insights into the Cyp51 isozyme selectivity of antifungal agents, we determined the MIC for 17 clinical antifungals and 13 pesticides against Δ*ku80,* Δ*cyp51A* and Δ*cyp51B* strains. The Δ*cyp51A* strain demonstrated 2- to 256-fold decreases in MICs for most azole antifungals (27/30) (Table 1). Fluconazole showed a 64-fold decrease, the highest among antifungal drugs, and difenoconazole showed a 256-fold decrease, the highest among all tested compounds, while MIC values of posaconazole, lanoconazole, and luliconazole were not altered. The increased drug susceptibility in the Δ*cyp51A* strain harboring only the Cyp51B isozyme suggests that most azole antifungal drugs are more selective against Cyp51B than against Cyp51A. The Δ*cyp51B* strain exhibited a more than fourfold reduction of MICs only for isavuconazole and prochloraz but exhibited increased MICs for isoconazole, miconazole, neticonazole, oxiconazole, and sulconazole (Table 1). These data indicate that Cyp51A primarily mediates the natural tolerance to most azole antifungals in *T. rubrum* and that different azoles may exhibit distinct selectivity for Cyp51 isozymes.

**TABLE 1:** MIC_100_ (minimum inhibitory concentration required to inhibit 100% of fungal growth) values (μg/mL) and MIC ratio (MIC of Δ*ku80*/MIC of Δ*cyp51A* or Δ*cyp51B*) of antifungal drugs and pesticides against Δ*ku80*, ∆*cyp51A*, and ∆*cyp51B* strains^*a*^

	MIC_100_ (μg/mL)			MIC of Δ*ku80*/MIC of Δ*cyp51A*	MIC of Δ*ku80*/MIC of Δ*cyp51B*
	Δ*ku80*	Δ*cyp51A*	Δ*cyp51B*		
Triazole					
Efinaconazole	0.02	0.005	0.01	4	2
Itraconazole	1	0.5	0.5	2	2
Ravuconazole	0.08	0.04	0.04	2	2
Fluconazole	32	0.5	16	64	2
Voriconazole	0.031	0.0078	0.016	4	2
Posaconazole	0.5	0.5	0.5	1	1
Isavuconazole	0.063	0.031	0.016	2	4
Imidazole					
Bifonazole	0.52	0.065	1	8	0.5
Clotrimazole	0.26	0.13	0.13	2	2
Isoconazole	0.26	0.13	0.52	2	0.5
Ketoconazole	1.4	0.18	1.4	8	1
Lanoconazole	0.00043	0.00043	0.00021	1	2
Luliconazole	0.00063	0.00063	0.00031	1	2
Miconazole	0.36	0.089	0.71	4	0.5
Neticonazole	0.0094	0.0047	0.019	2	0.5
Oxiconazole nitrate	0.1	0.026	0.21	4	0.5
Sulconazole	0.26	0.033	0.52	8	0.5
Imidazole pesticide					
Dimetconazole	0.2	0.0061	0.2	32	1
Difenoconazole	1.6	0.0061	1.6	256	1
Tebuconazole	1.6	0.024	0.78	64	2
Hexaconazole	1.6	0.012	0.78	128	2
Myclobutanil	3.1	0.098	3.1	32	1
Oxpoconazole	0.78	0.012	0.78	64	1
Triflumizole	3.1	0.024	3.1	128	1
Propiconazole	1.6	0.0061	1.6	128	1
Pefurazoate	0.2	0.1	0.1	2	2
Imazalil	0.13	0.0041	0.13	32	1
Prochloraz	0.26	0.13	0.032	2	8
Others					
Triforine	>100	25	>100	>4	ND^*b*^
Fenarimol	1.6	0.049	1.6	32	1

^*a*^ MIC values that were reproduced at least twice are shown in the table.

^*b*^ ND, not determined.

### Synergistic effects of azole combinations

Based on the different azole sensitivities of Δ*cyp51A* and Δ*cyp51B* strains, we explored the potential synergistic effects of azole combinations. We selected prochloraz, which demonstrated the highest fold changes in MIC values against Δ*cyp51B* strains, and fluconazole, sulconazole, and imazalil, which exhibited relatively high fold changes in MIC values, for the combination studies. In the WT strain, the combination resulted in fractional inhibitory concentration (FIC) indices of 0.32, 0.33, and 0.29 for the combination with fluconazole, sulconazole, and imazalil, respectively (Table 2), indicating synergistic effects. The Δ*ku80* strain demonstrated similar FIC indices (0.35, 0.28, and 0.31, respectively; Table 2), whereas the FIC indices for Δ*cyp51A* and Δ*cyp51B* strains were 1.0 and 0.62 for fluconazole, 0.81 and 0.78 for sulconazole, and 0.92 and 0.85 for imazalil, respectively (Table 2). These data suggest that dual azole combinations may represent a promising strategy for further investigation.

**TABLE 2:** Fractional inhibitory concentration (FIC) index of prochloraz combined with imazalil, fluconazole, or sulconazole against *Trichophyton rubrum* WT, parent strain Δ*ku80*, ∆*cyp51A*, and ∆*cyp51B^a^*

	FIC index		
	Fluconazole	Sulconazole	Imazalil
WT	0.33 ± 0.14 (n = 3)	0.29 ± 0.071 (n = 3)	0.32 ± 0.12 (n = 8)
Δ*ku80*	0.28 ± 0.13 (n = 2)	0.31 ± 0.13 (n = 2)	0.35 ± 0.035 (n = 4)
Δ*cyp51A*	1.0 ± 0.0 (n = 2)	0.81 ± 0.27 (n = 2)	0.92 ± 0.51 (n = 3)
Δ*cyp51B*	0.62 ± 0.0 (n = 2)	0.78 ± 0.31 (n = 2)	0.85 ± 0.28 (n = 3)

^*a*^ Mean ± SD.

To investigate whether the combination of azole antifungal drugs exerts a synergistic effect on other fungal species that possess Cyp51A and Cyp51B in their genome, we tested these combinations against *Aspergillus* sp., which also possesses two Cyp51 isozymes. The combinations of prochloraz with fluconazole, sulconazole, and imazalil were tested against the *Aspergillus welwitschiae* strains IFM 57545 (relatively azole-susceptible strain) and IFM 63877 (relatively azole-resistant strain). The FIC indices for the IFM 57545 and IFM 63877 strains were 0.41 and 0.38 for fluconazole, 0.44 and 0.25 for sulconazole, and 0.38 and 0.28 for imazalil, respectively (Table 3), suggesting the synergistic effects of these combinations. These data support the potential of this strategy against other pathogenic fungi.

**TABLE 3:** MIC values (μg/mL) ± SD and FIC index of prochloraz and imazalil combination in *A. welwitschiae* strains IFM 57545 and IFM 63897^*a*^

Strain	Agents	MIC (μg/mL)	FIC index with prochloraz
IFM 57545			
	Prochloraz	0.13 ± 0.12 (n = 7)	
	Fluconazole	23 ± 3.4 (n = 5)	0.41 ± 0.13 (n = 2)
	Sulconazole	0.13 ± 0.049 (n = 5)	0.44 ± 0.088 (n = 2)
	Imazalil	0.014 ± 0.0034 (n = 4)	0.38 ± 0.0 (n = 2)
IFM 63897			
	Prochloraz	0.18 ± 0.073 (n = 9)	
	Fluconazole	<300 (n = 3)	<0.38 (n = 3)
	Sulconazole	1.6 ± 0.47 (n = 4)	0.25 ± 0.088 (n = 2)
	Imazalil	0.064 ± 0.044 (n = 4)	0.28 ± 0.044 (n = 2)

^*a*^ Mean ± SD.

## DISCUSSION

This study demonstrated that the deletion of Cyp51 isozymes in *T. rubrum* significantly altered the organism’s susceptibility to azole antifungals. The differential MIC values observed in the Cyp51 isozyme deletion strains probably reflect the varying affinities of azole antifungals for these isozymes. This interpretation is consistent with previous biochemical studies in *A. fumigatus*, where recombinant protein analysis revealed that Cyp51A exhibits 3.0- to 37-fold higher dissociation constants (K_d_) with azole antifungals compared with those exhibited by Cyp51B (16). Although these findings are similar to our observations regarding the predominant role of Cyp51A in azole tolerance, direct biochemical studies using recombinant *T. rubrum* Cyp51 isozymes will be required to confirm similar binding properties in this species.

Using isozyme-deficient mutants, we demonstrated that Cyp51A and Cyp51B perform distinct roles in *T. rubrum*. Cyp51B is essential for basal growth, as evidenced by the severe growth inhibition observed in the Δ*cyp51B* strain. In contrast, Cyp51A is not essential for basal growth but is induced under azole antifungal treatment and promotes tolerance. This functional differentiation is remarkably different from observations in related fungi. For instance, the deletion of neither Cyp51A nor Cyp51B significantly affected mycelial growth in *A. fumigatus* (17). Similarly, *T. mentagrophytes*, a close relative of *T. rubrum*, exhibited minimal growth impairment after Cyp51B deletion (18). These contrasting findings suggest that the Cyp51 isozyme dependence of proliferation varies among species. These contrasting findings suggest that *T. rubrum* has evolved a greater dependence on Cyp51B for essential ergosterol biosynthesis, potentially reflecting its specialized adaptation to the keratinized environment of human skin and nails (19). This specialized niche may require more stringent control of sterol composition, making Cyp51B function more critical for survival compared to saprophytic or less host-adapted fungi.

The synergistic effects observed with combinations of azoles possessing different isozyme selectivities have significant therapeutic implications. This finding not only suggests novel treatment strategies for dermatophytosis but may also be applicable to infections caused by other fungi with multiple Cyp51 isozymes, including *Aspergillus*, *Fusarium*, and potentially *Mucor* species, where drug resistance remains problematic (20). The differential response to azoles after isozyme deletion is not unique to *T. rubrum*; similar variations in azole susceptibility between isozymes have been reported in *Fusarium* species (21–24). Although a previous study reported synergistic effects between antifungal azoles in some Mucorales isolates (25), the underlying mechanisms remained unclear. Our study suggests that isozyme selectivity is a key factor driving these synergistic interactions. We observed that a considerable number of compounds demonstrated strong selectivity for the Cyp51B isozyme. Conversely, a limited number of compounds exhibited high specificity for the Cyp51A isozyme. In future studies, it will be necessary to identify compounds with high specificity for Cyp51A, which may in turn result in the discovery of combinations of compounds that exert even stronger synergistic effects.

This study opens novel avenues for the development of antifungal drugs. The development of screening systems focused on isozyme specificity could identify azole combinations with improved antifungal activity. Importantly, our results may revitalize interest in compounds that were previously overlooked during drug development due to their apparent weak overall antifungal activity. Such compounds might have been overlooked not because they were inactive *per se*, but because their activity was isozyme-selective and thus masked in standard assays. These compounds may possess valuable isozyme-specific inhibitory properties that could be exploited in combination therapy approaches. While our *in vitro* findings demonstrate clear synergistic potential, our preliminary *in vivo* studies using fluconazole and prochloraz in a silkworm infection model (26) failed to demonstrate the synergistic effects observed *in vitro*. This discrepancy is likely due to the poor pharmacokinetic properties of prochloraz as an agricultural pesticide. Future studies should focus on identifying clinically suitable compounds with similar Cyp51A selectivity and conducting comprehensive *in vivo* efficacy evaluations to validate this combination therapy approach. Our findings reveal that Cyp51 isozyme selectivity represents a previously underexplored mechanism for antifungal synergy, providing a rational basis for precision combination therapies with enhanced efficacy against dermatophytosis and other fungal infections.

## MATERIALS AND METHODS

### Chemicals

Efinaconazole and luliconazole were purchased from BLD Pharmatech, Ltd., China. Ravuconazole, isavuconazole, and triforine were purchased from Merck, USA. Itraconazole, fluconazole, voriconazole, posaconazole, dimetconazole, ketoconazole, myclobutanil, difenoconazole, propiconazole, tebuconazole, hexaconazole, imazalil, and prochloraz were purchased from Tokyo Chemical Industry Co., Ltd., Japan. Oxpoconazole, clotrimazole, miconazole, triflunizole, pefurazoate, and fenarimol were purchased from FUJIFILM Wako Pure Chemical Corporation, Japan. Isoconazole and bifonazole were purchased from Thermo Fisher Scientific, Inc., USA. Sulconazole was purchased from Cayman Chemical Company, USA. Lanoconazole, neticonazole, and oxiconazole nitrate were purchased from MedChem Express, USA. All compounds were dissolved in dimethyl sulfoxide (DMSO); stock concentrations were adjusted and used so that the final concentration of DMSO used in the MIC assay was less than 1%.

### Fungal and bacterial strains and culture conditions

*T. rubrum* CBS118892 obtained from Westerdijk Fungal Biodiversity Institute was cultured on Sabouraud dextrose agar (SDA; 1% Bacto peptone, 4% glucose, 1.5% agar; pH after autoclaving was around 5.6) at 28°C. *A. welwitschiae* IFM 57545 and IFM 63877 strains were obtained from NBRP (27). Conidia were prepared as described previously (28). Briefly, *T. rubrum* was grown on a modified 1/10 Sabouraud dextrose agar (0.2% Bacto peptone, 0.1% glucose, 0.1% KH_2_PO_4_, 0.1% MgSO_4_ · 7H_2_O, 1.5% agar) at 27°C for 10–14 days, and a conidial suspension was prepared in sterile saline containing 0.05% (w/v) Tween 80. The solvent of the suspension was replaced with saline.

### Plasmid construction

To construct the *cyp51A*-targeting vector, pUC19Δ*cyp51A*, approximately 1.0 kb of the 5′-UTR fragments of the *cyp51A* open reading frame (ORF) was amplified from *T. rubrum* genomic DNA by PCR. The *neomycin phosphotransferase* gene cassette, which consists of *Escherichia coli* neomycin phosphotransferase (*nptII*), *A. nidulans trpC* promoter (*PtrpC*), and *A. fumigatus cgrA* terminator (*TcgrA*) with the 5′-UTR fragments of the *cyp51A* ORF, was amplified from pAg1-*cyp51A*-3′-UTR (15) using the primer pair *PtrpC*-F and *cyp51A*-3′-R-pUC19. The plasmid backbone of pUC19 was cleaved using *Kpn*I/PstI. These three fragments were joined using the In-Fusion HD Cloning Kit (TaKaRa Bio, Japan).

To construct the *cyp51B*-targeting vector, pUC19Δ*cyp51B*, approximately 1.4 and 1.1 kb of the 5′- and 3′-UTR fragments of the *cyp51B* ORF were amplified from *T. rubrum* genomic DNA. The plasmid backbone of pUC19 was cleaved using *Kpn*I/PstI. The *neomycin phosphotransferase* gene cassette, which consists of *nptII*, *PtrpC*, and *TcgrA*, was amplified from pAg1-ΔTr*cla4* (29). These fragments were joined using the In-Fusion HD Cloning Kit. The primers used in this study are listed in Table S1.

### Transformation of *T. rubrum*

*T. rubrum* was transformed using the polyethylene glycol (PEG) method as described previously (30, 31). The desired transformants and purified genomic DNA were evaluated by PCR. Total DNA was extracted using the Quick-DNA Fungal/Bacterial Miniprep Kit (Zymo Research, USA). Fungal cells were triturated using μT-01 (TAITEC, Japan) using 5 mm stainless beads.

### Antifungal susceptibility assay

MICs were determined according to the broth microdilution method of the Clinical and Laboratory Standards Institute. Conidia (2 × 10^3^) were incubated with twofold serial dilutions of antifungal agents in 200 µL of MOPS-buffered RPMI (pH 7.0) using 96-well microtiter plates at 28°C for 1 day (for *A. welwitschiae*) or 7 days (for *T. rubrum*), and MIC_100_ (minimum inhibitory concentration required to inhibit 100% of fungal growth) was determined visually (32). We performed two technical replicates to measure the MIC. If the value was reproducible, we listed that value as the MIC. If the value was different between the first and second attempts, we made further attempts, and the value confirmed more than once was listed as the MIC in Table 1.

The FIC index was calculated using the following formula: FIC index = (MIC_Acom_/MIC_A_) + (MIC_Bcom_/MIC_B_), where MIC_Acom_ and MIC_Bcom_ are the MIC_100_ of drugs tested in combination, and MIC_A_ and MIC_B_ are the MIC_100_ of drugs tested alone. Synergy was defined as an FIC index of ≤0.5 (33). Each experiment was performed at least twice.

### Quantitative reverse transcription-PCR (qRT-PCR)

Total RNAs were purified using NucleoSpin RNA (Macherey-Nagel) and reverse-transcribed into cDNAs using ReverTra Ace (Toyobo) according to the manufacturers’ instructions. qRT-PCR was performed using TB Green Premix Ex Taq II (TaKaRa Bio, Japan) on a StepOne Real-Time PCR System (Thermo Fisher Scientific, USA). The relative mRNA expression level was determined using the 2^−∆∆Ct^ method using *Chitin synthase I* (*chs1*) as an endogenous control to normalize the samples (31). The primers used in this study are listed in Table S1.

### Statistics

The mean values of the mycelial growth diameter and Cyp51 mRNA expression levels were compared using one-way analysis of variance with Tukey’s post-hoc test using Prism 9 (GraphPad, USA). Differences were considered significant at *P* < 0.05.

## Data Availability

The data that support the findings of this study are available from the corresponding author upon reasonable request.

## SUPPLEMENTAL MATERIAL

Fig. S1 aac.00598-25-s0001.tiff Mycelial growth of Δku80, ∆cyp51B, and an independently generated ∆cyp51B strain.

Table S1 aac.00598-25-s0002.pdf Primers used in this study.

ASM does not own the copyrights to Supplemental Material that may be linked to, or accessed through, an article. The authors have granted ASM a non-exclusive, world-wide license to publish the Supplemental Material files. Please contact the corresponding author directly for reuse.
