# Supplementary figures and images for: Synergistic effects of Cyp51 isozyme-specific azole antifungal agents on fungi with multiple cyp51 isozyme genes
Source: Antimicrob Agents Chemother. 2025 Sep 26;69(11):e00598-25. doi: 10.1128/aac.00598-25 (PMC12587598; doi:10.1128/aac.00598-25)

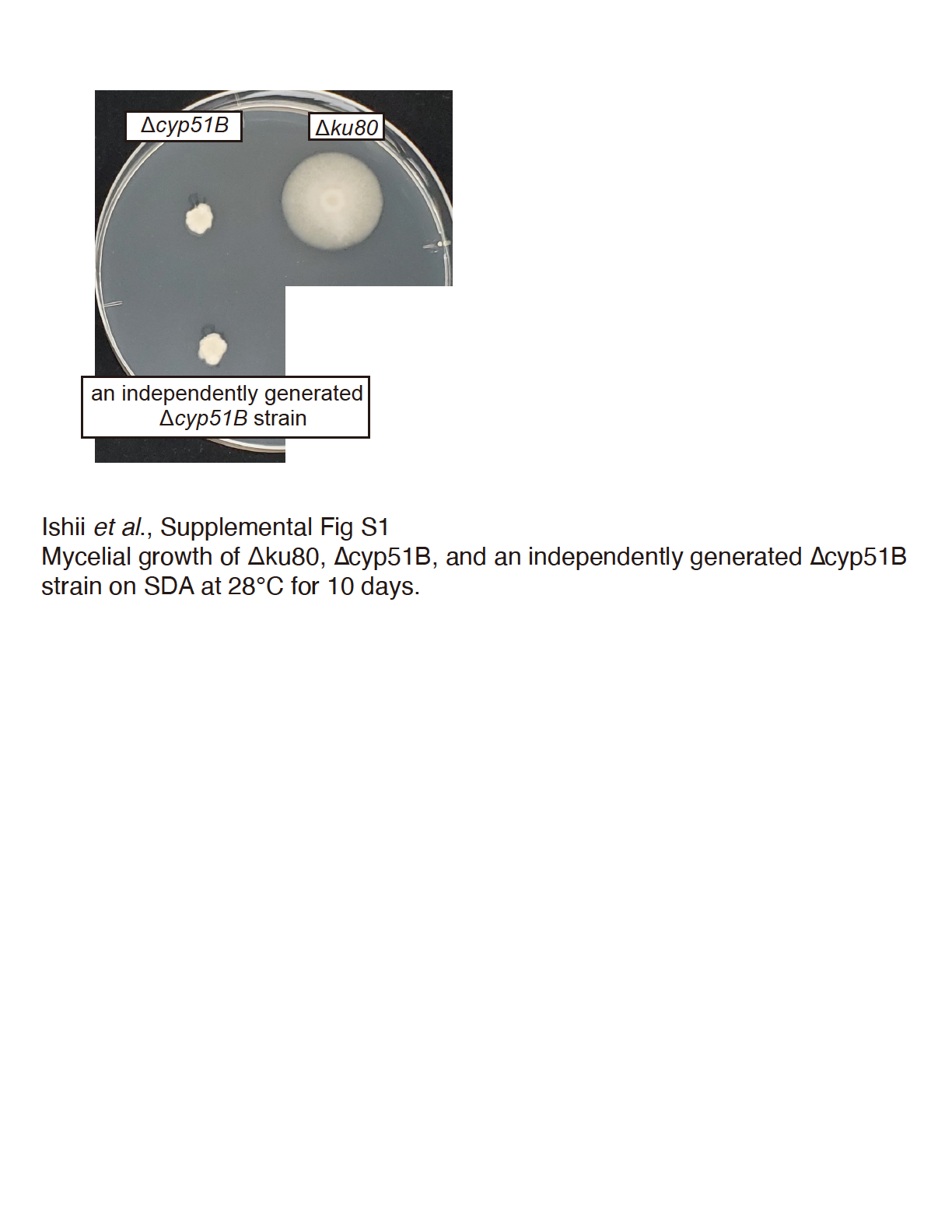

Supplement: Fig. S1 — Mycelial growth of Δku80, ∆cyp51B, and an independently generated ∆cyp51B strain. [file aac.00598-25-s0001.tiff]
