# Supplementary material for: Synergistic effects of Cyp51 isozyme-specific azole antifungal agents on fungi with multiple cyp51 isozyme genes
Source: Antimicrob Agents Chemother. 2025 Sep 26;69(11):e00598-25. doi: 10.1128/aac.00598-25 (PMC12587598; doi:10.1128/aac.00598-25)
Supplement: Table S1 — Primers used in this study. [file aac.00598-25-s0002.pdf]

Table S1 Primers used in this study.

Primer name	Sequences
cyp51A-5′-F-pUC19	5′-CCAAGCTTGCATGCCTGCTCAGGCCCAATTCTCTG-3′
cyp51A-5′-R-*PtrpC*	5′-TCAATATCATCTTCTTTGAAGAAATGTTGGCGGCG-3′
cyp51A-3′-R-pUC19	5′-GTGAATTCGAGCTCGAGCTCGGAAATGCCTTGACA-3′
cyp51B-5′-F-pUC19	5′-CGAGCTCGAATTCACCTCTACATCTGCCTCTGCGG-3′
cyp51B-5′-R	5′-CAATATCATCTTCTGCTGCCTCTTCTTCTGCTGCT-3′
cyp51B-3′-F	5′-ATGAATCATCTATCGTCACGATTGCGGATTGAATC-3′
cyp51B-3′-R-pUC19	5′-TCAATATCATCTTCTGCGTACGGTACCTTCACCAA-3′
PtrpC-F	5′-AGAAGATGATATTGAAGGAGCACTTTTTGGGCTT-3′
TcgrA-R	5′-AGATGATTCATGACGTATATTCACCG-3′
chs1-RT-F	5′-GGCCACAACGAAGCCTATGA-3′
chs1-RT-R	5′-CAATCGGCCTGGGAGATG-3′
cyp51A-RT-F (Primer 1)	5′-GTGGGTCCCTTTGCCACATA-3′
cyp51A-RT-R (Primer 2)	5′-TTGGACTTAGCTCCTTCGCG-3′
cyp51B-RT-F (Primer 3)	5′-GAACAACGTTGGTGTCACCG-3′
cyp51B-RT-R (Primer 4)	5′-ACATCTGTGTCTGCCTGAGC-3′
